# Sequential Field Therapy in Actinic Keratosis: A Mechanism-Based Rationale for Complementary Treatment Strategies

**DOI:** 10.3390/jcm15124553

**Published:** 2026-06-11

**Authors:** Giulio Gualdi, Gabriele Soligon, Patrick Silvetti, Leonardo Balestra, Davide Bertolla, Luca Fania, Francesco Ricci, Mario Puviani, Paolo Sbano, Andrea Paradisi

**Affiliations:** 1 University Hospital, Ospedale Maggiore di Parma, 43126 Parma, Italy; giulio.gualdi@unipr.it (G.G.); gabriele.soligon@unipr.it (G.S.); patrick.silvetti@unipr.it (P.S.); leonardo.balestra@unipr.it (L.B.); 2 Section of Dermatology and Infectious Diseases, Department of Medical Sciences, University of Ferrara, 44121 Ferrara, Italy; 3 Skin Cancer Center, Istituto Dermopatico dell’Immacolata-IRCCS, 00167 Rome, Italy; 4 Department of Life Science, Health, and Health Professions, Link University of Rome, 00168 Rome, Italy; 5 Dermatology Clinic, Sassuolo Hospital, 41049 Sassuolo, Italy; 6 Dermatology Unit, Department of Medical Sciences, Surgery and Neurosciences, University of Siena, 53100 Siena, Italy; 7 Dermatology Unit, Department of Medical, Surgical, Abdominal and Endocrine-Metabolic Sciences, Fondazione Policlinico Universitario A. Gemelli IRCCS, 00168 Rome, Italy

**Keywords:** actinic keratosis, field cancerization, sequential treatment, tirbanibulin, 5-fluorouracil, diclofenac, imiquimod, photodynamic therapy

## Abstract

**Background:** Actinic keratoses are common keratinocytic precursor lesions arising within chronically ultraviolet-damaged skin and are associated with an increased risk of progression to cutaneous squamous cell carcinoma. The concept of field cancerization has shifted therapeutic strategies from the treatment of isolated visible lesions toward broader field-directed approaches targeting both clinical and subclinical disease. **Methods:** This narrative review summarizes the rationale, mechanisms of action, efficacy profile, tolerability, and practical limitations of currently available field-directed therapies for actinic keratosis, including 5-fluorouracil, imiquimod, diclofenac, photodynamic therapy, and tirbanibulin. Based on their distinct biological targets, we propose a mechanism-based framework for sequential treatment strategies. **Results:** Available therapies act through partially non-overlapping mechanisms, including cytotoxic activity, immune activation, cyclooxygenase-2 inhibition, photodynamic oxidative damage, and tubulin/Src pathway inhibition. These complementary effects provide a biological rationale for sequential regimens aimed at addressing the heterogeneity of field cancerization. However, direct clinical evidence supporting specific treatment sequences remains limited, and proposed regimens should be interpreted as hypothesis-generating rather than as validated therapeutic protocols. **Conclusions:** Mechanism-based sequential field therapy may represent a rational strategy to optimize long-term control of actinic keratosis and field cancerization. Prospective comparative studies are needed to define optimal sequences, treatment intervals, patient selection criteria, and clinically meaningful endpoints, including sustained field clearance, recurrence reduction, tolerability, adherence, and prevention of progression to invasive cutaneous squamous cell carcinoma.

## 1. Introduction

Actinic keratosis (AK) is a common keratinocytic lesion caused by chronic ultraviolet (UV) radiation and is recognized as a precursor to invasive cutaneous squamous cell carcinoma (cSCC) [1]. It represents a major dermatological and oncological concern, particularly in aging populations with cumulative sun exposure. Although the annual malignant transformation risk of a single AK is relatively low, the cumulative risk in patients with multiple lesions and extensive field damage is clinically significant. Reported progression rates vary widely (0.01–10% per lesion every year) [2]. Risk increases with lesion thickness, degree of dysplasia, lesion size and immunosuppression [3]. The concept of field cancerization, originally described by Slaughter et al., refers to areas of chronically sun-damaged skin containing genetically altered keratinocytes predisposed to multiple primary tumors [4]. Because AK progression involves multiple oncogenic processes—including uncontrolled proliferation, impaired apoptosis, immune evasion, and a pro-tumorigenic microenvironment—single-agent therapy may not fully eradicate the diseased field. Sequential field-directed treatments combining non-overlapping mechanisms of action may therefore enhance clearance and reduce recurrence risk. Within this framework, the present review examines the main field-directed therapies currently used for AKs, focusing on their pharmacological targets, clinical role, efficacy, tolerability, and practical limitations. Building on the available evidence for individual agents, we then outline a mechanism-based framework for complementary treatment sequencing in field cancerization. The proposed sequences are intended to provide a biologically grounded rationale for future clinical studies and should be interpreted as hypotheses to be tested, rather than as validated therapeutic algorithms.

## 2. Field-Directed Therapies

### 2.1. Topical 5-Fluorouracil

Topical 5-fluorouracil (5-FU) represents one of the most extensively validated field-directed therapies for actinic keratoses (AKs) and cutaneous field cancerization. AKs develop on chronically ultraviolet-damaged skin and are increasingly considered part of a biological continuum of keratinocyte carcinogenesis, sharing key molecular alterations with cSCC, including mutations involving TP53 and NOTCH signaling pathways that drive clonal expansion of dysplastic keratinocytes [5]. Subclinical lesions within the cancerization field may outnumber clinically visible AKs by up to tenfold and harbor similar genetic alterations, supporting therapeutic strategies targeting the entire photodamaged area rather than isolated lesions [5]. 5-FU acts as a pyrimidine analog that irreversibly inhibits thymidylate synthase, disrupting DNA synthesis and selectively inducing apoptosis in rapidly proliferating atypical keratinocytes. Beyond its antimetabolite activity, 5-FU also exerts immunomodulatory effects by reducing immunosuppressive myeloid-derived suppressor cells and promoting immunogenic cell death through release of damage-associated molecular patterns (DAMPs), thereby enhancing local anti-tumor immune surveillance within the cancerization field [6]. Supported by randomized controlled trials and current American Academy of Dermatology recommendations, topical 5-FU is strongly endorsed as a first-line field therapy capable of targeting both clinical and subclinical disease [7,8]. Recent reports have also explored topical 5-FU-based regimens as cytoreductive strategies in selected keratinocyte carcinomas, further supporting the broader clinical versatility of this therapeutic class [9,10].

### 2.2. Imiquimod

Topical imiquimod represents a key immune-mediated field-directed therapeutic option for the management of AKs within the framework of cutaneous cancerization fields. Acting as an immune response modifier, imiquimod activates innate and adaptive immune pathways through stimulation of toll-like receptor-7 signaling, leading to interferon-α and pro-inflammatory cytokine production that promotes recruitment of cytotoxic T lymphocytes and immune-mediated apoptosis of dysplastic keratinocytes within the cancerization field [11]. This mechanism enables treatment of both clinically visible lesions and subclinical atypical clones distributed throughout the cancerization field. Treatment is frequently associated with a transient increase in lesion count resulting from the unmasking of previously subclinical AKs—defined as the maximum lesion count (Lmax)—which reflects pharmacologically induced detection and subsequent immune-mediated clearance of field disease [12]. Comparative evidence from recent meta-analyses indicates that, although photodynamic therapy—particularly 5-aminolevulinic acid photodynamic therapy (ALA-PDT)—may achieve higher lesion clearance rates, imiquimod remains an effective field therapy providing sustained immune-mediated control of actinic damage, albeit with more frequent local inflammatory reactions reflecting treatment activity [11]. Consistent with these findings, current American Academy of Dermatology guidelines recommend topical imiquimod as an established field-directed treatment capable of reducing lesion burden across contiguous areas of actinically damaged skin [7].

### 2.3. Diclofenac

Topical diclofenac sodium 3% formulated in a hyaluronic acid vehicle represents a well-established therapeutic option for the management of AKs, particularly in patients with limited disease burden or in clinical contexts requiring treatments characterized by favorable tolerability and self-administration. Diclofenac exerts its activity primarily through inhibition of cyclooxygenase-2 (COX-2), leading to reduced prostaglandin E_2_ synthesis, a key mediator implicated in ultraviolet-induced keratinocyte carcinogenesis and maintenance of the tumor-promoting microenvironment. Suppression of prostaglandin signaling has been associated with decreased cellular proliferation, inhibition of angiogenesis, and restoration of apoptosis susceptibility in dysplastic keratinocytes, resulting in progressive regression of lesions in sun-damaged skin [7]. Clinical responses typically develop gradually during prolonged treatment courses, reflecting this modulatory rather than directly cytotoxic mechanism of action. In a recent multicenter randomized controlled trial comparing diclofenac 3% gel with potassium hydroxide 5% solution and placebo, complete clearance at the end of treatment was achieved in 23.8% of patients treated with diclofenac, confirming modest but clinically meaningful efficacy together with a favorable safety profile dominated by mild localized skin reactions [13]. Although characterized by a slower onset of action compared with more inflammatory field therapies, long-term outcomes demonstrated converging efficacy during follow-up, supporting diclofenac as a low-intensity anti-inflammatory field- or lesion-directed strategy particularly suited for early or mild AK presentations and for patients requiring sustained therapy with limited treatment-related inflammation.

### 2.4. PDT

Photodynamic therapy (PDT) represents an established physician-directed field therapy for the treatment of AKs, particularly suitable for patients presenting with multiple lesions within chronically photodamaged skin. PDT involves topical application of a photosensitizing precursor, most commonly 5-aminolevulinic acid (ALA) or methyl aminolevulinate (MAL), which is metabolized through the heme biosynthetic pathway into intracellular protoporphyrin IX (PpIX). Preferential accumulation of PpIX in dysplastic keratinocytes results from altered cellular metabolism and reduced ferrochelatase activity. Upon illumination with visible light, activation of PpIX generates reactive oxygen species inducing oxidative cellular injury, mitochondrial dysfunction, apoptosis, autophagy, and immunogenic cell death across the cancerization field [14,15].

Randomized controlled trials have demonstrated high lesion clearance rates while enabling treatment of both clinically visible and subclinical lesions over contiguous areas of actinic damage [7]. PDT can therefore be employed as both lesion-directed and field-directed therapy and may contribute to delaying development of new lesions within the cancerization field [14]. Emerging protocols including daylight, short-incubation, and simulated daylight PDT have demonstrated comparable efficacy with markedly reduced treatment-associated pain, significantly improving patient tolerability and facilitating broader clinical adoption [15].

### 2.5. Tirbanibulin

Topical tirbanibulin 1% ointment represents a recently introduced field-directed therapy for AKs, characterized by a novel mechanism of action and an abbreviated treatment course. Tirbanibulin is a first-in-class antiproliferative agent that inhibits tubulin polymerization and disrupts Src kinase signaling, inducing p53 upregulation, cell-cycle arrest at the G2/M phase, and caspase-mediated apoptosis in proliferating dysplastic keratinocytes while limiting extensive inflammatory damage within surrounding actinically damaged skin [16]. Clinical efficacy was demonstrated in two identically designed randomized phase III trials including 702 patients with AKs of the face or scalp treated once daily for five consecutive days, achieving pooled complete clearance rates of approximately 49% compared with 9% with vehicle at day 57, together with predominantly mild and transient local skin reactions and no treatment discontinuations related to adverse events [16]. Subsequent focused American Academy of Dermatology guideline updates issued a strong recommendation supported by high-certainty evidence for topical tirbanibulin as a field-directed therapy, emphasizing the favorable balance between efficacy, tolerability, and feasibility associated with the short-course regimen [17,18]. The molecular targets, efficacy profiles, typical dosing regimens, and practical treatment burden of the main field-directed therapies are summarized in Table 1, Table 2 and Table 3.

## 3. Mechanism-Based Sequential Treatment Framework

The introduction of the concept of the cancerization field has led to a paradigm shift in the treatment of AKs, where it is necessary to treat not only discrete clinically observable lesions but also entire skin areas where mutagenic processes are presumed to have already occurred due to actinic damage, albeit still in a subclinical form. Thus, there has been a transition from the exclusive use of physical therapies such as cryotherapy and laser therapy, or even surgical excision, which still play a fundamental role in many clinical settings, to the use of topical pharmacological therapies [7,8,11,13,14,15,16,17,18]. These drugs, in various formulations designed to improve the transepidermal delivery of the active molecule, allow for the treatment of large sections of skin biologically affected by the carcinogenesis process due to UV radiation, serving both a curative role in frankly pathological lesions and in perilesional skin areas that are damaged but not yet clinically evaluable, although they are already genetically altered [4,5]. This would not be feasible with traditional therapies. Moreover, the availability of different drugs enables clinicians to target AKs through various biochemical pathways, achieving a potentially complementary effect that underpins a sequential approach to treating the cancerization field, which, by its diffuse and chronic nature, often requires multiple treatments to achieve adequate disease control [7,8,11,13,14,15,16,17,18]. This is the exact goal of the article: to hypothesize, based on the known mechanisms of action of the different molecules, what the best sequences for drug use might be to optimize their effectiveness. Below, we propose potential treatment sequences in the form of a sequential triple therapy, along with the rationale behind each of them.

### 3.1. Sequential Treatments Starting with 5-FU

(THE SO-CALLED “CYTOTOXIC DEBULKING”)

Such treatment sequences are identified as the most suitable choices in cases of significant solar damage with extensive cancerization fields and numerous AKs that have proven resistant to traditional physical therapies such as cryotherapy [5,7,8].

#### 3.1.1. 5-FU → IMIQUIMOD → DICLOFENAC

Initial cytotoxic elimination of proliferating dysplastic keratinocytic clones exerted by 5-FU is then followed by immune-mediated clearance of residual mutated cells performed by IMIQUIMOD applications and subsequent stabilization of the cancerization microenvironment via COX-2 suppression obtained by DICLOFENAC action [6,11,13].

Following a descending gradient of effectiveness in clearing mutated keratinocyte clones, the initial tumor debulking is maximized, subsequent immunological surveillance is strengthened, and long-term control of the cancerization field is maintained.

#### 3.1.2. 5-FU → PDT → TIRBANIBULIN

In this sequence, the focus is primarily on the dysplastic clones with high mitotic activity, characterized by elevated proliferative and metabolic activity, through the inhibition of DNA synthesis operated by 5-FU, the oxidative metabolic destruction of photodynamic therapy, and the blockade of the mitotic spindle catalyzed by tirbanibulin [6,14,15,16].

### 3.2. Sequential Treatments Starting with IMIQUIMOD

(THE SO-CALLED “IMMUNE PRIMING”)

In this case, the initial treatment with imiquimod aims at immune activation and strengthening the immunological surveillance of the cancerization field [11,12].

#### 3.2.1. IMIQUIMOD → 5-FU → TIRBANIBULIN

IMIQUIMOD stimulates antigen presentation to enhance the activation of the immune system and the recognition of dysplastic keratinocytes by itself; the subsequent cytotoxic action of 5-FU and the antiproliferative effect of TIRBANIBULIN target any residual disease [6,11,16].

#### 3.2.2. IMIQUIMOD → PDT → DICLOFENAC

The activation of the immune system is combined through the Toll-like receptor (TLR-7) of antigen-presenting cells thanks to the action of IMIQUIMOD, the cellular destruction mediated by reactive oxygen species due to the effect of PDT, and the final anti-inflammatory stabilization of the cancerization field with prolonged application of DICLOFENAC [11,13,14,15].

This therapeutic option can be used in cases of extensive disease or for patients who present lower disease burden.

### 3.3. Sequential Treatments Starting with DICLOFENAC

(THE SO-CALLED “MICROENVIRONMENT MODULATION)

Prolonged treatment with diclofenac allows for control of the cancerization field when the disease burden is low [7,13]. Subsequent therapies, while more effective in clearing more advanced AKs but associated with higher rates of local adverse reactions, enable the removal of more resistant and stubborn precancerous lesions that were unresponsive to initial therapy.

#### 3.3.1. DICLOFENAC → 5-FU → IMIQUIMOD

The reduction in inflammation and angiogenesis mediated by the COX-2 inhibition of DICLOFENAC would increase the susceptibility of dysplastic cells to cytotoxic therapies (5-FU) and immune-mediated therapies (IMIQUIMOD) [6,7,11,13].

#### 3.3.2. DICLOFENAC → PDT → TIRBANIBULIN

This involves control of the microenvironment (DICLOFENAC) followed by oxidative destruction through one or more sessions of PDT and proliferative blockade of residual dysplastic cells by TIRBANIBULIN [13,14,15,16].

### 3.4. Sequential Treatments Starting with Photodynamic Therapy

Initially, PDT is performed to achieve good disease control in a few sessions, especially for patients with low therapeutic compliance [14,15].

#### 3.4.1. PDT → 5-FU → IMIQUIMOD

PDT reduces the disease burden, 5-FU eliminates surviving keratinocyte clones, and the application of IMIQUIMOD provides long-term immuno-mediated control [6,11,14,15].

#### 3.4.2. PDT → TIRBANIBULIN → DICLOFENAC

A few sessions of PDT are performed to achieve adequate initial disease control, followed by a short-term regimen with TIRBANIBULIN to eliminate surviving proliferating dysplastic cells, and control of the inflammatory microenvironment through applications of DICLOFENAC for several weeks [13,14,15,16].

### 3.5. Sequential Treatment Starting with Tirbanibulin

A rapid clearance of dysplastic cells is achieved through proliferative blockade with moderate local adverse reactions, and subsequently, more or less aggressive therapeutic regimens are chosen based on the disease burden [16,17,18].

#### 3.5.1. TIRBANIBULIN → 5-FU → IMIQUIMOD

A rapid proliferative arrest is achieved through the action of TIRBANIBULIN, followed by a cytotoxic phase (5-FU) and an immune-mediated phase (IMIQUIMOD) [6,11,16].

#### 3.5.2. TIRBANIBULIN → PDT → DICLOFENAC

Application of TIRBANIBULIN for 5 days accounts for a proliferative arrest of dysplastic keratinocytes through inhibition of the mitotic spindle, leading to ROS-mediated destruction of surviving dysplastic clones and final stabilization of the inflammatory microenvironment [13,14,15,16]. The proposed mechanism-based treatment sequences are summarized in Table 4.

Overall, these proposed sequences should be interpreted as mechanism-based hypotheses rather than validated clinical protocols [7,17,18]. Their clinical value, optimal timing, and comparative efficacy require confirmation in prospective studies.

## 4. Practical Considerations

### 4.1. Addressing Field Heterogeneity

A cancerization field involves keratinocyte clones at different stages of dysplasia, with heterogeneous proliferative activity, mutational burden, and immune visibility [4,5]. Sequential therapy targeting distinct oncogenic pathways may increase the probability of eliminating both clinically visible and subclinical lesions. Future studies should therefore combine clinical lesion counts with standardized field-severity scores and, when available, non-invasive imaging endpoints to assess both visible and subclinical disease.

### 4.2. Reduction of Therapeutic Resistance

Repeated use of a single mechanism (e.g., only cytotoxic therapy) may leave resistant cellular subpopulations. Combining the following may reduce clonal survival and recurrence risk through non-overlapping biological pressure [6,11,13,14,15,16]:

- Cytotoxic effects (5-FU, PDT);
- Immune-mediated clearance (IMIQUIMOD);
- Antiproliferative signaling inhibition (TIRBANIBULIN);
- Microenvironment modulation (DICLOFENAC).

### 4.3. Personalized Treatment Planning

Sequential regimens may be tailored according to

- Lesion burden and thickness;
- Anatomical site;
- Patient age and immune status;
- Tolerability and adherence profile.

For example, frail or elderly patients may benefit from short-course regimens (TIRBANIBULIN, PDT) integrated with immune or anti-inflammatory phases, whereas high-risk immunosuppressed patients might require cytotoxic–immune combinations for maximal oncologic control [6,7,11].

### 4.4. Potential Role in Secondary Cancer Prevention

The strongest evidence for reductions in subsequent cSCC development currently exists for 5-FU field therapy [7,8]. Sequential integration with immune modulators may theoretically enhance long-term tumor surveillance, though this remains to be validated in prospective trials.

## 5. Limitations

### 5.1. Limited Direct Clinical Evidence

Most available data derive from studies evaluating single agents, not structured sequences [7,17,18]. The proposed regimens are therefore based on mechanistic rationale and indirect comparative evidence rather than randomized sequence trials. Moreover, mechanistic complementarity should not be assumed to imply clinical synergy, as sequential use may also lead to pharmacodynamic interference depending on timing, inflammatory status, and residual proliferative activity.

### 5.2. Cumulative Toxicity

Sequential inflammation from cytotoxic or immune therapies may reduce adherence or increase treatment discontinuation [7,11,15,16].

Careful spacing between treatment phases and patient education are essential. Transition to the next treatment phase should therefore be individualized according to clinical response, resolution of local inflammation, patient tolerability, and treatment goals.

### 5.3. Lack of Standardized Protocols

Optimal sequencing, intervals between treatments, and total treatment duration remain undefined [7,17,18].

Heterogeneity in AK grading and outcome measures further complicates comparisons across studies. Future studies should define treatment intervals, rest periods, criteria for proceeding to the next phase, supportive care measures, and predefined stop or hold rules.

### 5.4. Economic and Practical Considerations

Multi-step regimens increase healthcare utilization and cost, which may limit real-world feasibility.

### 5.5. AK Severity Scores and Instrumental Evaluation of Efficacy

In the literature, several scoring systems have been proposed to evaluate the severity and extent of AK, including the AKASI (Actinic Keratosis Area and Severity Index), AK-FAS (Actinic Keratosis Field Assessment Scale), the Investigator’s Global Assessment (IGA), and the total lesion count. However, the presence of multiple evaluation methods makes it challenging to objectively compare the efficacy of different therapeutic approaches across studies.

Moreover, clinical improvement or apparent clearance of lesions does not necessarily correspond to complete histological resolution. This discrepancy between clinical and histopathological outcomes further complicates the assessment of treatment effectiveness and the comparison of different therapies [19]. At the same time, histological evaluation of treatment outcomes is often difficult to perform in routine clinical practice due to ethical considerations, since repeated biopsies may not always be justified or acceptable for patients. For these reasons, non-invasive imaging technologies of the new generation, such as reflectance confocal microscopy (RCM), line-field confocal optical coherence tomography (LC-OCT), high-frequency ultrasound, and other emerging diagnostic tools, may play an increasingly important role. These techniques could help provide a more objective and reproducible assessment of treatment response, allowing clinicians and researchers to better evaluate the real clinical and subclinical outcomes of different therapies for AKs [20]. A minimal standardized outcome set for future studies could include lesion count, a validated field-severity score, a predefined follow-up timepoint, patient-reported tolerability, and, when feasible, an imaging-based assessment of subclinical clearance.

## 6. Future Perspectives

The future management of AKs is expected to evolve toward a precision-medicine framework integrating advances in molecular biology, digital diagnostics, and preventive dermatology. Increasing evidence supports the concept that AK represents a heterogeneous manifestation of cutaneous cancerization fields, in which clinically visible lesions coexist with genetically altered but subclinical keratinocyte clones distributed across chronically photodamaged skin. This biological complexity underscores the need for therapeutic strategies capable of targeting both individual lesions and the broader carcinogenic field, while simultaneously addressing the molecular pathways driving keratinocyte dysplasia and progression toward cSCC [21,22]. One promising area of development concerns novel immune-modulating and targeted topical therapies. Agents such as resiquimod, a dual toll-like receptor-7/8 agonist, have demonstrated encouraging results in early clinical trials, achieving high lesion clearance rates through enhanced activation of innate and adaptive immune responses [23,24]. In parallel, investigational molecules targeting angiogenesis, cell proliferation, and inflammatory pathways—including potassium dobesilate, betulinic acid derivatives, and nanoparticle-based antiproliferative drugs—are being explored to interfere with the molecular mechanisms underlying keratinocyte carcinogenesis [25,26]. Although these agents remain largely investigational, they highlight the ongoing transition toward mechanism-driven treatment strategies. Recent translational studies have begun to clarify the molecular landscape underlying AK development and progression. Recurrent alterations involving genes regulating keratinocyte differentiation, DNA damage repair, and tumor suppression—particularly TP53, NOTCH signaling pathways, and inflammatory mediators induced by ultraviolet radiation—have been identified as key drivers of early keratinocyte carcinogenesis. These discoveries are fostering the search for molecular biomarkers capable of predicting lesion behavior, identifying high-risk patients, and guiding individualized treatment strategies. In the near future, biomarker-driven risk stratification may allow clinicians to distinguish indolent lesions from those with a higher probability of progression to invasive cSCC, thereby improving both therapeutic decision-making and long-term surveillance strategies [5]. Parallel advances in artificial intelligence (AI) and digital dermatology are expected to significantly enhance the diagnostic and monitoring capabilities in AK [27]. Recent studies evaluating image-recognizing multimodal artificial intelligence models in actinic keratosis/squamous cell carcinoma, melanoma versus nevi, and basal cell carcinoma versus common mimickers highlight their potential as adjunctive diagnostic tools, while also underscoring the need for prospective validation before routine clinical use [28,29,30]. AI-assisted image analysis may also enable automated lesion detection and objective quantification of treatment outcomes. When integrated with non-invasive imaging modalities—including reflectance confocal microscopy, line-field confocal optical coherence tomography, and high-frequency ultrasound—AI-based tools could facilitate earlier identification of subclinical disease within the cancerization field and allow more precise monitoring of therapeutic response in both clinical trials and routine practice [27]. This may be particularly relevant in AK, where apparent surface clearance may not fully reflect persistence of subclinical field damage. At the therapeutic level, the next generation of AK treatments is likely to focus on mechanism-driven targeted therapies and immune modulation. Novel topical agents under investigation include immune stimulators such as resiquimod, antiproliferative compounds targeting keratinocyte mitotic pathways, and molecules capable of modifying the tumor-promoting inflammatory microenvironment. In addition, optimization of existing modalities—such as daylight photodynamic therapy, shortened incubation protocols, and improved photosensitizers—aims to maintain high efficacy while significantly reducing treatment-related discomfort and improving patient adherence [15]. These developments reflect a broader shift toward therapies that are both biologically targeted and patient-centered. Equally important is the expanding emphasis on preventive dermatology and chemoprevention. Given the chronic and recurrent nature of AK, prevention strategies remain essential to reduce the incidence of new lesions and the cumulative risk of keratinocyte carcinoma. Regular photoprotection, early detection programs, and patient education remain the cornerstone of preventive interventions. In addition, pharmacologic prevention targeting inflammatory or carcinogenic pathways—such as systemic retinoids or anti-inflammatory agents—is being explored in high-risk populations, including immunosuppressed individuals and organ transplant recipients. Overall, the future landscape of AK management will likely be characterized by the convergence of molecular diagnostics, AI-assisted clinical evaluation, targeted therapeutic strategies, and preventive approaches. This integrated model of care has the potential to transform AK management from a largely lesion-oriented approach into a comprehensive strategy aimed at controlling cancerization fields, improving long-term disease control, and ultimately reducing the burden of invasive cSCC.

## 7. Conclusions

Mechanism-based sequential therapy represents a biologically rational strategy to manage cancerization fields by integrating cytotoxic, immune-mediated, antiproliferative, photodynamic, and microenvironment-targeted effects. However, whether such approaches improve clearance durability or reduce progression to invasive cSCC remains unproven. Prospective controlled studies are needed to validate specific sequences and define optimal combinations, timing, patient selection, and clinically meaningful endpoints.

## Figures and Tables

**Table 1:** Molecular targets and mechanisms of action of topical and field-directed therapies used in the management of actinic keratosis and cutaneous cancerization field. The table summarizes the principal molecular pathways targeted by currently available therapies and their downstream cellular effects contributing to clearance of clinically visible and subclinical dysplastic keratinocytes.

*Therapy*	Primary Molecular Target	Key Molecular Mechanism	Cellular Effects	Effect on Field Cancerization
***5-Fluorouracil***	Thymidylate synthase	Inhibition of thymidine synthesis and incorporation into RNA/DNA	DNA damage, S-phase arrest, apoptosis of rapidly proliferating keratinocytes; immunogenic cell death	Eradication of clinical and subclinical dysplastic clones
***Imiquimod***	Toll-like receptor 7 (TLR7)	Activation of innate immune signaling with IFN-α, TNF-α and cytokine release	Recruitment of cytotoxic T cells and NK cells; immune-mediated apoptosis	Immune clearance of atypical keratinocytes across the field
***Diclofenac (3% HA gel)***	Cyclooxygenase-2 (COX-2)	Inhibition of prostaglandin E_2_ synthesis	Reduced proliferation, angiogenesis inhibition, restoration of apoptosis susceptibility	Modulation of tumor-promoting inflammatory microenvironment
***Tirbanibulin (1%)***	Tubulin polymerization; Src kinase signaling	Disruption of microtubule assembly and Src-dependent proliferation pathways	G2/M arrest, p53 upregulation, caspase-mediated apoptosis	Targeted antiproliferative elimination of dysplastic keratinocytes
***Photodynamic therapy (ALA/MAL-PDT)***	Intracellular protoporphyrin IX (PpIX) activation	Light-induced generation of reactive oxygen species	Oxidative damage, mitochondrial dysfunction, vascular injury, apoptosis and immunogenic cell death	Destruction of visible and subclinical lesions within the cancerization field

**Table 2:** Comparative efficacy and typical dosing regimens of field-directed therapies. Complete clearance rates are approximate ranges derived from heterogeneous studies using different formulations, endpoints, treatment areas, and assessment timepoints; they should not be interpreted as direct head-to-head comparisons. Dosing schedules refer to commonly used or approved regimens and may vary according to formulation, anatomical site, disease burden, and local prescribing recommendations.

Treatment	Mechanism	Typical Regimen and Application Frequency	Complete Clearance (%)	Follow-Up
*5-FLUOROURACIL*	CYTOTOXIC	ONCE OR TWICE DAILY DEPENDING ON FORMULATION; COMMONLY 2–4 WEEKS	70–90	12 MONTHS OR MORE
*IMIQUIMOD*	IMMUNOMODULATOR	FORMULATION-DEPENDENT, E.G., 5% TWICE WEEKLY FOR 16 WEEKS, OR 3.75% ONCE DAILY IN TWO 2-WEEK CYCLES SEPARATED BY A 2-WEEK TREATMENT-FREE INTERVAL	60–75	12 MONTHS OR MORE
*DICLOFENAC*	COX-2 INHIBITION	TWICE DAILY FOR 60–90 DAYS	30–60	6–12 MONTHS
*PDT (ALA/MAL)*	PHOTODYNAMIC	PHYSICIAN-ADMINISTERED PROTOCOL; USUALLY 1–2 TREATMENT SESSIONS, DEPENDING ON PHOTOSENSITIZER, LIGHT SOURCE, AND CLINICAL RESPONSE	70–90	12–24 MONTHS
*TIRBANIBULIN*	TUBULIN/ SRC INHIBITION	ONCE DAILY FOR 5 CONSECUTIVE DAYS	44–54	9–12 MONTHS

**Table 3:** Treatment adherence and practical burden of field-directed therapies. Adherence is influenced not only by treatment duration and local skin reaction severity, but also by cosmetic downtime, need for clinic attendance, treatment-area restrictions, cost/accessibility, and patient preference. The categories reported below are qualitative and should be interpreted as practical considerations rather than as comparative efficacy endpoints.

Treatment	Duration	Adherence	Main Limiting Factors
*5-FLUOROURACIL*	LONG	MODERATE–LOW	PROLONGED VISIBLE INFLAMMATION, ERYTHEMA, CRUSTING, EROSIONS, BURNING/STINGING, COSMETIC DOWNTIME, AND POSSIBLE INTERFERENCE WITH DAILY OR SOCIAL ACTIVITIES
*IMIQUIMOD*	LONG	MODERATE	PROLONGED INTERMITTENT REGIMEN; ERYTHEMA, SCALING, CRUSTING, EROSION OR ULCERATION, PRURITUS OR BURNING; POSSIBLE TREATMENT INTERRUPTIONS; OCCASIONAL FLU-LIKE SYSTEMIC SYMPTOMS
*DICLOFENAC*	VERY LONG	LOW	SLOW ONSET OF RESPONSE, LOWER COMPLETE CLEARANCE COMPARED WITH MORE INTENSIVE FIELD THERAPIES, AND CUMULATIVE ADHERENCE BURDEN
*PDT*	SHORT	HIGH	PROCEDURE-RELATED PAIN OR DISCOMFORT, NEED FOR CLINIC-BASED TREATMENT AND EQUIPMENT, POST-TREATMENT ERYTHEMA/CRUSTING, PHOTOSENSITIVITY PRECAUTIONS, AND ACCESS/COST CONSIDERATIONS
*TIRBANIBULIN*	VERY SHORT	VERY HIGH	COST/ACCESS CONSIDERATIONS, RESTRICTED TREATMENT-FIELD SIZE, LIMITED LONG-TERM FIELD-CONTROL DATA, AND RECURRENCE AFTER INITIAL COMPLETE CLEARANCE DESPITE GENERALLY MILD-TO-MODERATE LOCAL REACTIONS

**Table 4:** A summary of the expected biological effects of different treatment sequences, chosen based on the initial agent and the complementarity of mechanisms.

*Starting Agent*	Sequence	Mechanistic Complementarity	Expected Biological Effect
*5-FLUOROURACIL*	5-fluorouracil → imiquimod → diclofenac	Cytotoxic → immune → anti-inflammatory	Debulking + immune clearance + field stabilization
	5-fluorouracil → PDT → tirbanibulin	DNA synthesis inhibition → ROS → mitotic/Src blockade	Multilevel tumor cell destruction
*IMIQUIMOD*	Imiquimod → 5-FU → tirbanibulin	Immune priming → cytotoxic → antiproliferative	Enhanced immune-mediated tumor control
	Imiquimod → PDT → diclofenac	Immune → oxidative → anti-inflammatory	Broad field effect, good tolerability
*DICLOFENAC*	Diclofenac → 5-FU → imiquimod	Microenvironment modulation → cytotoxic → immune	Reduced pro-tumor signaling before eradication
	Diclofenac → PDT → tirbanibulin	Anti-inflammatory → ROS → mitotic blockade	Sequential control of field carcinogenesis
*PDT*	PDT → 5-FU → imiquimod	ROS debulking → cytotoxic → immune	High clearance + immune surveillance
	PDT → tirbanibulin → diclofenac	ROS → antiproliferative → anti-inflammatory	Clearance with reduced recurrence signals
*TIRBANIBULIN*	Tirbanibulin → 5-FU → imiquimod	Mitotic arrest → cytotoxic → immune	Stepwise intensification
	Tirbanibulin → PDT → diclofenac	Growth arrest → ROS → field stabilization	Lower inflammatory burden

## Data Availability

No new data were created or analyzed in this study.
